# Supplementary material for: Two-dimensional single-crystalline mesoporous high-entropy oxide nanoplates for efficient electrochemical biomass upgrading
Source: Nat Commun. 2024 Aug 8;15:6761. doi: 10.1038/s41467-024-50721-2 (PMC11310307; doi:10.1038/s41467-024-50721-2)
Supplement: Supplementary file 1 — Supplementary Information [file 41467_2024_50721_MOESM1_ESM.pdf]

Supplementary Information

Two-Dimensional Single-Crystalline Mesoporous High-Entropy Oxide Nanoplates for Efficient Electrochemical Biomass Upgrading

Yanzhi Wang,[1] Hangjuan He,[1] Hao Lv,[1,2] Fengrui Jia,[1] and Ben Liu[1,*]

[1]Key Laboratory of Green Chemistry and Technology of Ministry of Education, College of Chemistry, Sichuan University, Chengdu 610064, China. E-mail: ben.liu@scu.edu.cn

[2]School of Chemistry and Chemical Engineering, Shanghai Jiao Tong University, Shanghai 200240, China

Materials and Methods

Materials and Chemicals. Urea, oleic acid, n-hexane, and ethanol were obtained from Sigma-Aldrich. Cobalt nitrate hexahydrate, nickel nitrate hexahydrate, manganese nitrate tetrahydrate, copper nitrate trihydrate, zinc nitrate hexahydrate, iron nitrate nonahydrate, and bismuth nitrate pentahydrate were purchased from the Aladdin. All the reagents were utilized as received without further purification.

Extended synthesis of SC-MHEO. Other SC-MHEO nanoplates were synthesized by a similar route, but changing the kinds and concentrations of metal ions in solution B. **For SC-MHEO-$(CoNiMnCuFe)_3O_4$**, 0.60 mmol of cobalt nitrate, 0.50 mmol of nickel nitrate, 0.30 mmol of manganese nitrate, 0.20 mmol of copper nitrate and 0.20 mmol of ferric nitrate were added to 10 mL of deionized water to form solution B. **For SC-MHEO-$(CoNiMnCuZnBi)_3O_4$**. 0.55 mmol of cobalt nitrate, 0.40 mmol of nickel nitrate, 0.25 mmol of manganese nitrate, 0.20 mmol of copper nitrate, 0.20 mmol of zinc nitrate and 0.20 mmol of bismuth nitrate were added to 10 mL of deionized water to form solution B. **For SC-MHEO-$(CoNiMnCuZnFeBi)_3O_4$**, 0.55 mmol of cobalt nitrate, 0.30 mmol of nickel nitrate, 0.20 mmol of manganese nitrate, 0.20 mmol of copper nitrate, 0.20 mmol of zinc nitrate, 0.20 mmol of ferric nitrate and 0.20 mmol of bismuth nitrate were added to 10 mL of deionized water to form solution B. **For SC-MHEO-(CoNiMnCuZn)O,** calcination process was carried out under N_2 atmosphere.

Synthesis of P-HEO. P-HEO was prepared by the reported high-temperature methods (Angew. Chem. Int. Ed. 2021, 60, 20253). An alkaline medium solution with a pH of 10 was prepared by adding 25 mL of ammonia (NH_3, Merck, 25%) to 200 mL of deionized water. Subsequently, a total amount of 0.10 M metal precursor was added to the above solution. The molar ratio of elements is: Co:Mn:Ni:Cu:Zn=4.3:3.5:2.0:1.6:1.0. The mixture was stirred continuously at room temperature for 1 h. After the filtration, the precipitate was dried at 60°C for 4 h and gently ground evenly in an agate mortar. Finally, it was calcined at 950°C for 1 h to obtain P-HEO.

Catalyst ink preparation. 5.0 mg of electrocatalyst was ultrasonically dispersed in a mixed solution of 0.33 mL of ultrapure water, 0.16 mL of ethanol, and 10 µL of Nafion. 0.20 mL of resulting catalyst ink was dropped onto 1 cm^{-2} of carbon paper, followed by natural drying within a culture dish with a cover. The loading is 2.0 mg/cm^{-2}. A compact film was formed and used as the working electrode in the following electrolysis process.

HPLC analysis. HPLC (FULI Instruments LC-5090 system, China) with a C18 column (4.6 mm×250 mm SinoChrom ODS-BP 5 µm) and an ultraviolet-visible (UV-Vis) detector were used to examine the products of HMF electrooxidation. The test process is as follows: 50 µL of electrolyte solution was taken after constant potential electrolysis, diluted to 5.0 mL with deionized water, and analyzed by HPLC. The wavelength of UV-

Vis detector was set at 265 nm. Mobile phases A and B were methanol and 5.0 mM ammonium formate aqueous solution, respectively. The volume ratio of A/B was 3:7. The flow rate is 1.0 mL min^{-1}.

Density Functional Theory (DFT) calculations. Our calculations were performed by means of DFT+U methods using the Vienna Ab-initio Simulation Package (VASP)[1-3]. The electron-ion interactions were described by using projector augmented wave (PAW) method[4, 5]. The exchange and correlation energies were determined with the Perdew, Burke, and Ernzerhof (PBE) functional with the on-site Coulomb Repulsion U term was used[6, 7]. In this work, U(Co)= 3.0 eV, U(Ni)= 5.5 eV, U(Mn)= 4.0 eV, and U(Zn)= 4.7 eV for electronic structure calculations. The cutoff energy for the plane wave-basis expansion was set to 400 eV. The convergence criterion of electronic structure was set to 10^{-4} eV, and the atomic relaxation was continued until the forces acting on atoms were smaller than 0.05 eV/Å. The Brillouin zone was sampled with the k-point mesh with the separation of 0.04 $Å^{-}$. A Gaussian smearing of 0.05 eV was applied to speed up electronic convergence. A vacuum height of 15 Å along the vertical direction was selected to avoid the unwanted interaction between the slab and its period images. The final structure was illustrated with VESTA software[8]. The computational hydrogen electrode (CHE) model was used to calculate the reaction pathway, and the potential of reversible hydrogen electrode (RHE) was selected as the the reference potential[9]. The chemical potential of the proton-electron pair was defined as half-gaseous hydrogen molecule chemical potential.

Characterization. SEM images were collected using a JEOL JSM-7600F field emission Scanning Electron Microscope. SEM samples were prepared by dropcasting a suspension of the sample powder onto a silicon wafer. TEM and STEM studies were carried out using a field emission TEM (JEM-F200, JEOL Ltd., Japan) with an accelerating voltage of 200 kV. TEM and STEM samples were prepared by dropcasting a diluted suspension of the sample powder onto a carbon coated copper grid (300 mesh). Powder X-ray Diffraction (XRD) patterns were recorded using a D/max 2500 VL/PC diffractometer (Japan) equipped with graphite-monochromatized Cu Kα radiation in 2ϑ ranging from 30° to 90°. The working voltage and current were 40 kV and 100 mA, respectively. X-ray photoelectron spectroscopic (XPS) spectra were performed on a scanning X-ray microprobe (Thermo ESCALAB 250Xi) that uses Al Kα radiation. The binding energy of the C 1s peak (284.8 eV) was used as a standard to calibrate the binding energies of other elements.

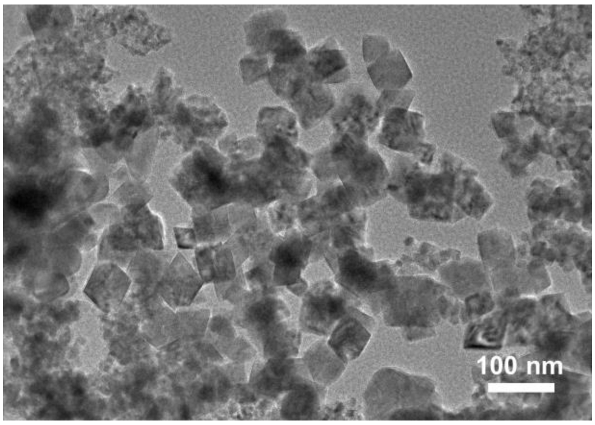

Supplementary Figure 1. SEM image of P-HEO-$(CoNiMnCuZn)_3O_4$ with disordered structure and morphology.

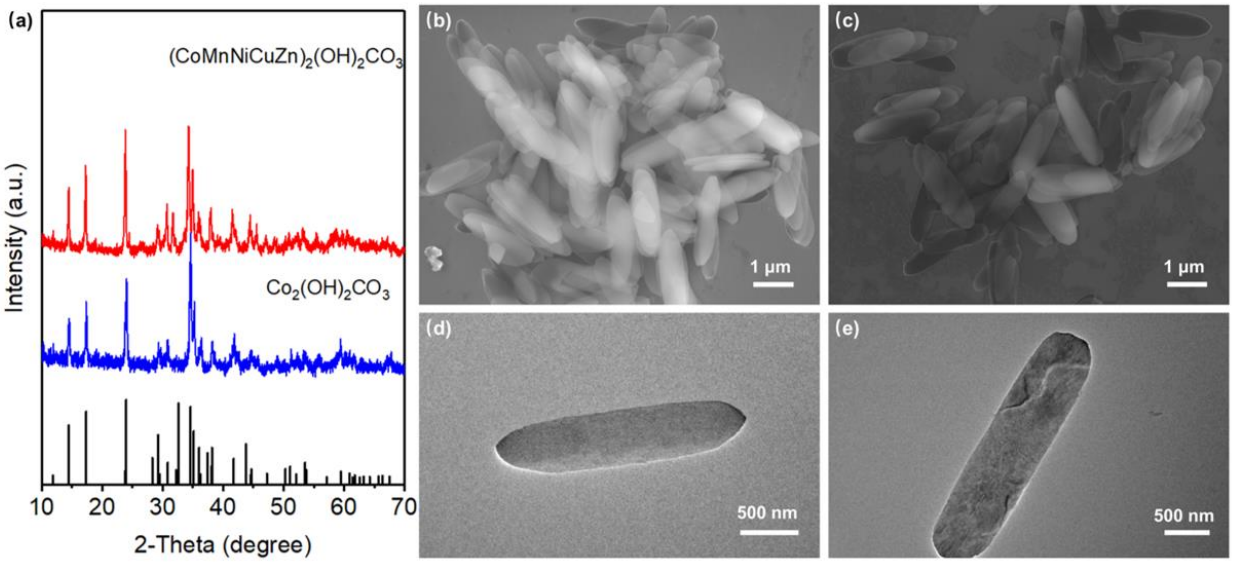

Supplementary Figure 2. (a) XRD patterns of $(CoMnNiCuZn)_2(OH)_2CO_3$ and $Co_2(OH)_2CO_3$. (b,c) SEM and (d,e) TEM images of (b,d) $(CoMnNiCuZn)_2(OH)_2CO_3$ and (c,e) $Co_2(OH)_2CO_3$.

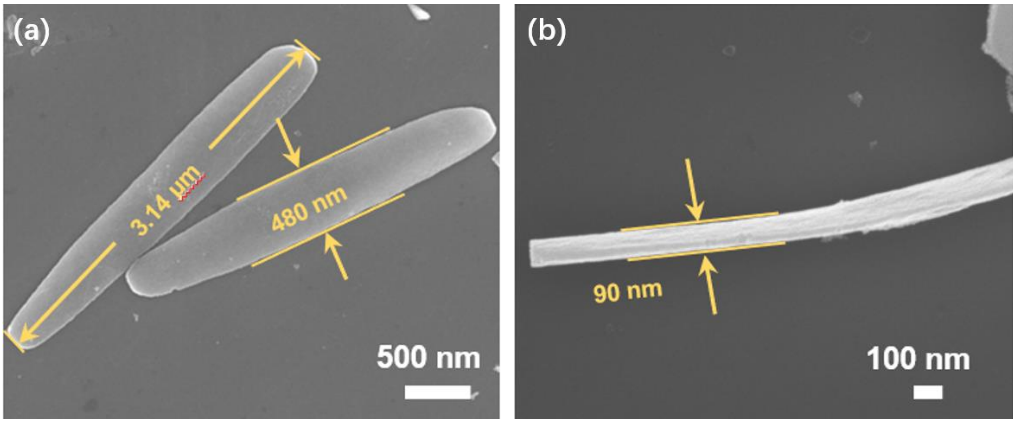

Supplementary Figure 3. SEM images of $(CoMnNiCuZn)_2(OH)_2CO_3$ observed from (a) top and (b) side views.

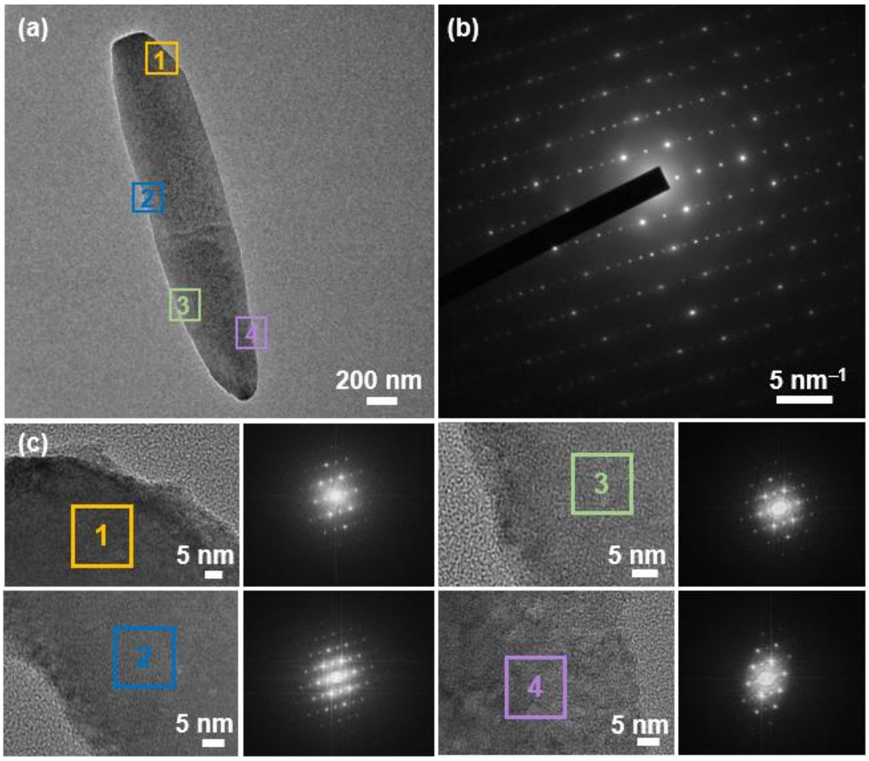

Supplementary Figure 4. (a) TEM image and (b) corresponding SAED pattern, and (c) high-resolution TEM images and corresponding FT patterns of $(CoNiMnCuZn)_2(OH)_2CO_3$.

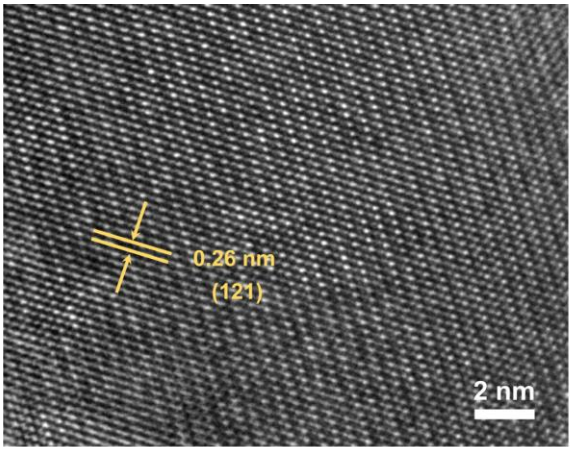

Supplementary Figure 5. High-resolution TEM image of $(CoNiMnCuZn)_2(OH)_2CO_3$.

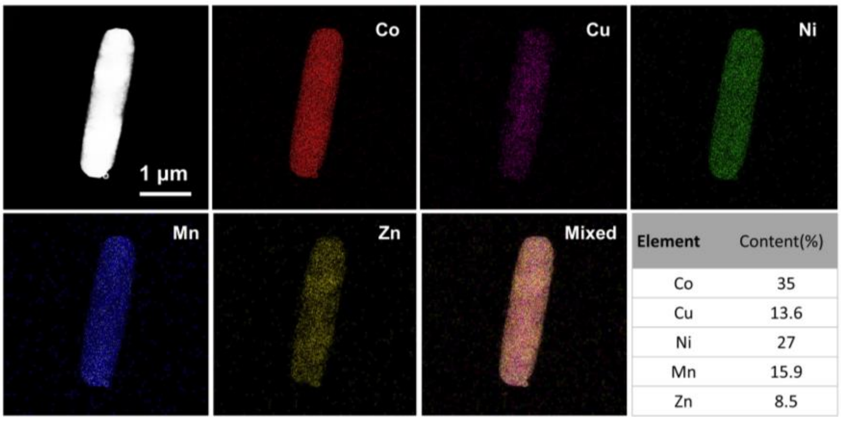

Element	Content(%)
Co	35
Cu	13.6
Ni	27
Mn	15.9
Zn	8.5

Supplementary Figure 6. HAADF-STEM EDS mapping images and corresponding element ratio of two-dimensional $(CoMnNiCuZn)_2(OH)_2CO_3$ nanoplates.

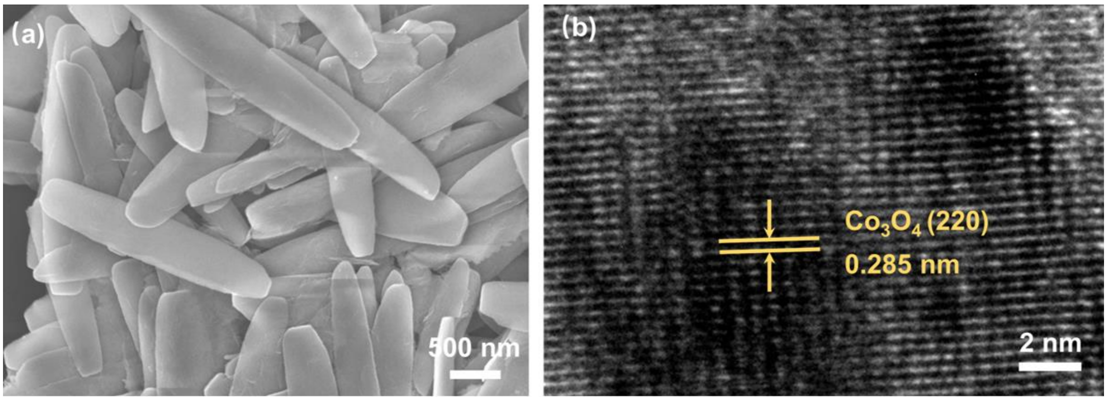

Supplementary Figure 7. (a) SEM and (b) high-resolution TEM images of SC-M-Co_3O_4.

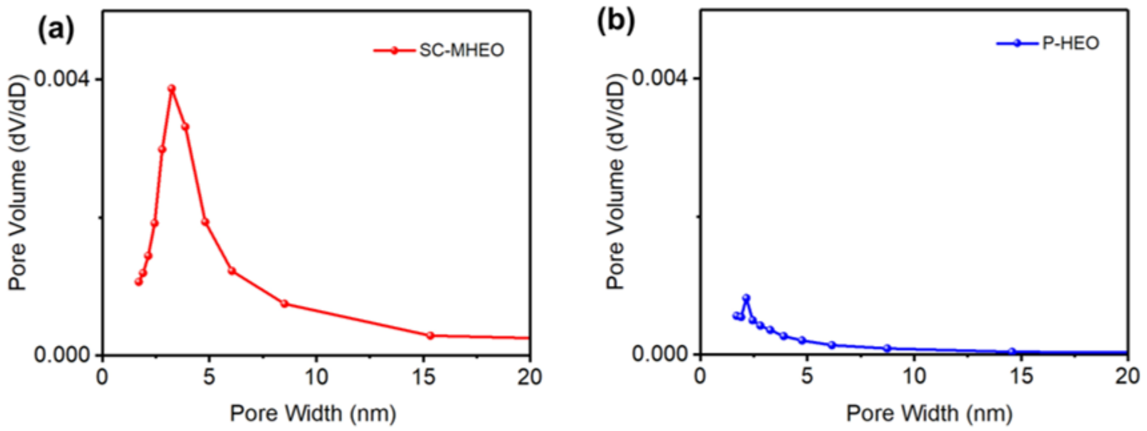

Supplementary Figure 8. Pore size distributions of (a) SC-MHEO and (b) P-HEO summarized from N_2 sorption isotherms.

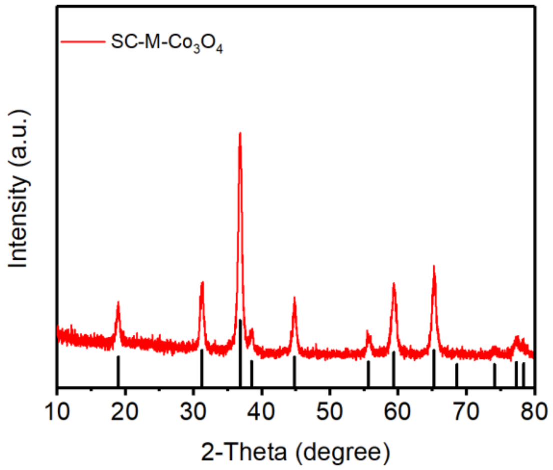

Supplementary Figure 9. Wide-angel XRD pattern of SC-M-Co_3O_4.

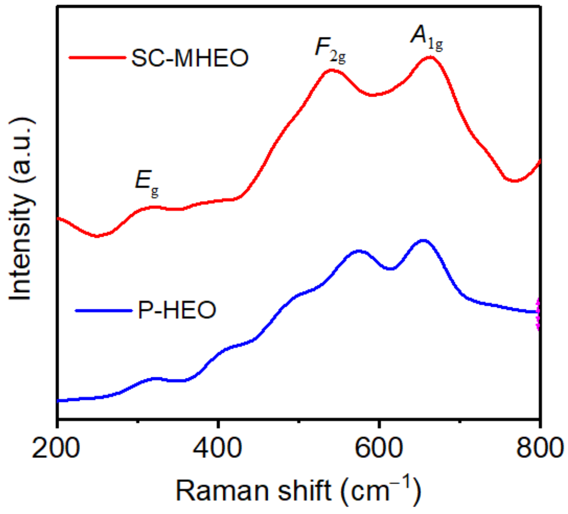

Supplementary Figure 10. Raman spectra of SC-MHEO and P-HEO.

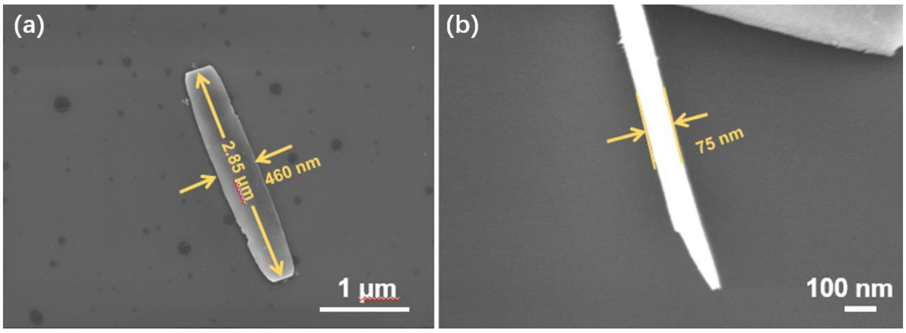

Supplementary Figure 11. SEM images of SC-MHEO observed from (a) top and (b) side views.

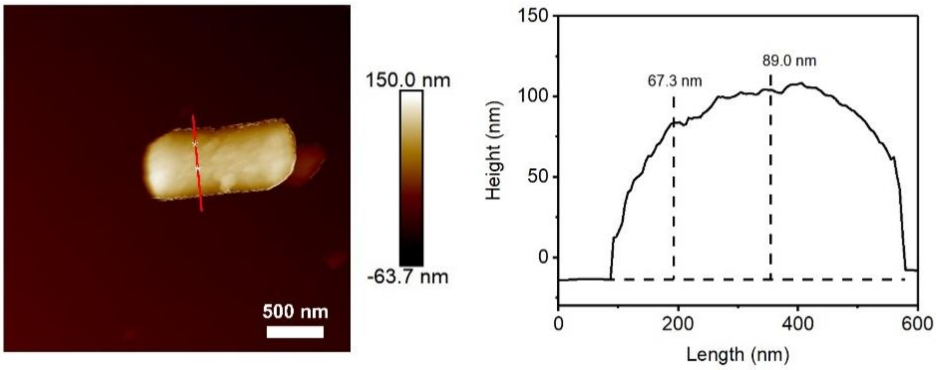

Supplementary Figure 12. AFM image of SC-MHEO and height profile along the red line in AFM.

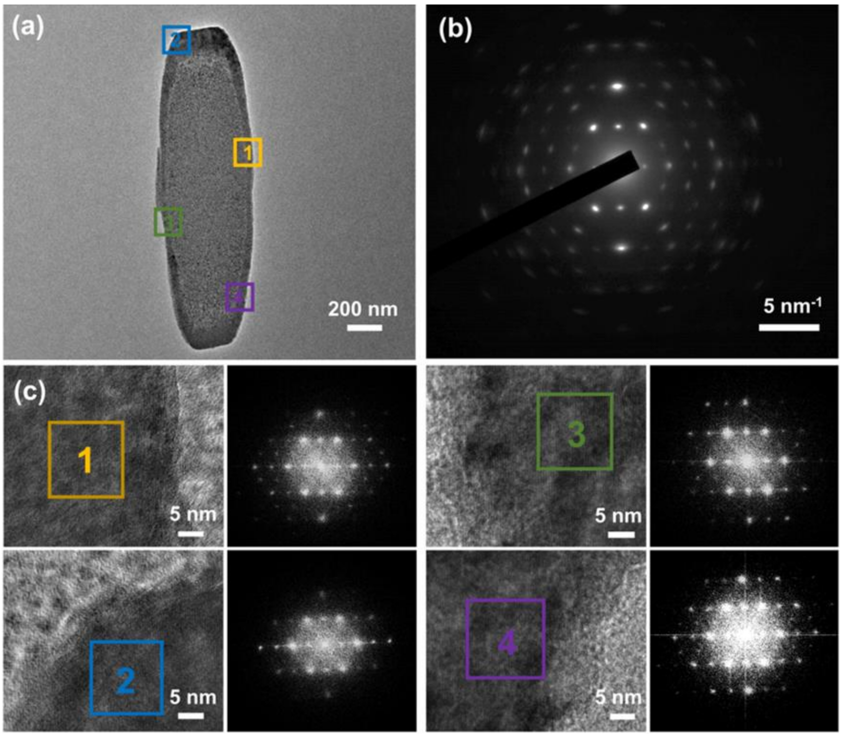

Supplementary Figure 13. (a) TEM image and (b) corresponding SAED pattern, (c) high-resolution TEM images and corresponding FT patterns of SC-M-Co_3O_4.

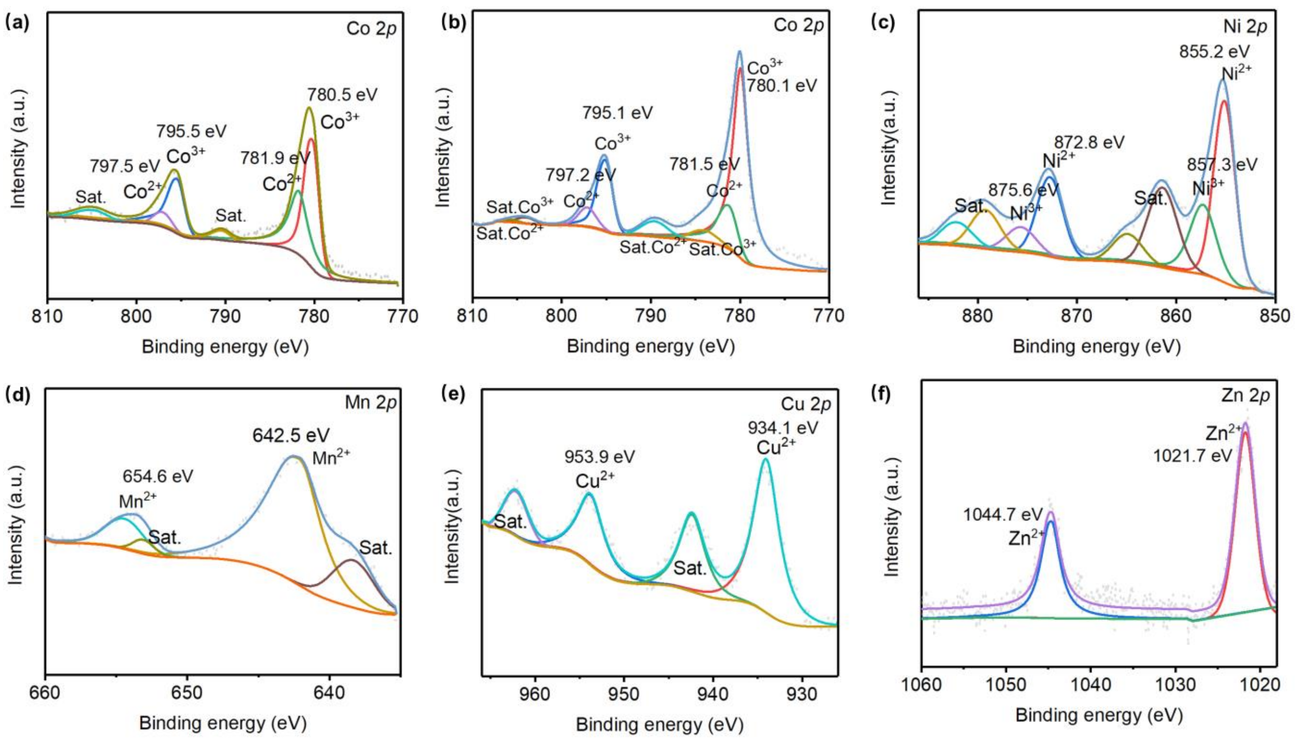

Supplementary Figure 14. (a) High-resolution XPS spectra of Co 2*p* for SC-M-Co_3O_4. High-resolution XPS spectra of (b) Co 2*p*, (c) Ni 2*p*, (d) Mn 2*p*, (e) Cu 2*p*, and (f) Zn 2*p* for SC-MHEO-$(CoNiMnCuZn)_3O_4$.

Note for Supplementary Figure 14: In the Co XPS spectrum of SC-M-Co_3O_4, the Co $2p^{3/2}$ peaks located at 780.5 eV and 781.9 eV are attributed to Co^{3+} and Co^{2+}, respectively. The ratio of Co^{3+}/Co^{2+} is 1.82. In the XPS spectrum of SC-MHEO, the Ni $2p^{3/2}$ peaks located at 855.2 eV and 857.3 eV are attributed to Ni^{2+} and Ni^{3+}, respectively. The Mn $2p^{3/2}$ and Mn $2p^{1/2}$ peaks located at 642.5 eV and 654.6 eV are attributed to Mn^{2+}. The Cu $2p^{3/2}$ and Cu $2p^{1/2}$ peaks located at 934.1 eV and 953.9 eV are attributed to Cu^{2+}. The Zn $2p^{3/2}$ and Zn $2p^{1/2}$ peaks located at 1021.7 eV and 1044.7 eV are attributed to Zn^{2+}.

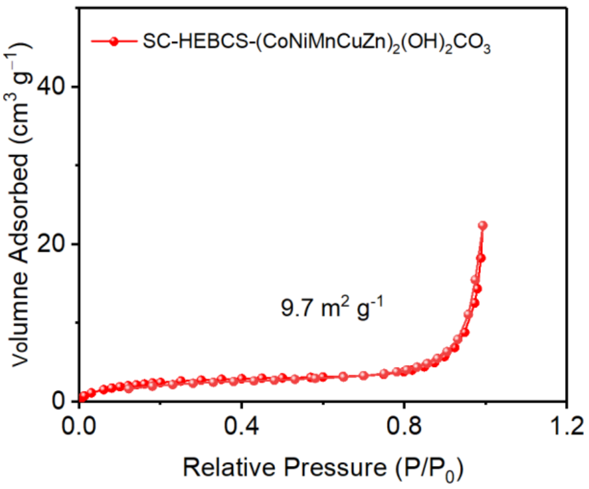

Supplementary Figure 15.N_2 sorption isotherms of BCS-$(CoNiMnCuZn)_2(OH)_2CO_3$.

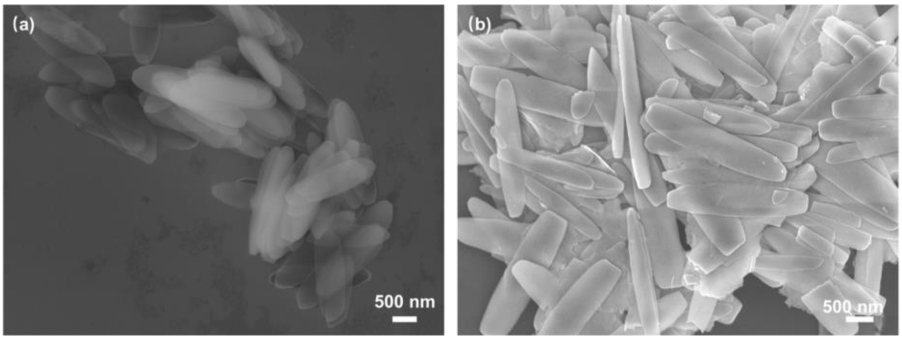

Supplementary Figure 16. SEM images of (a) $(CoMnNiCuFe)_2(OH)_2CO_3$ and (b) SC-MHEO-$(CoMnNiCuFe)_3O_4$.

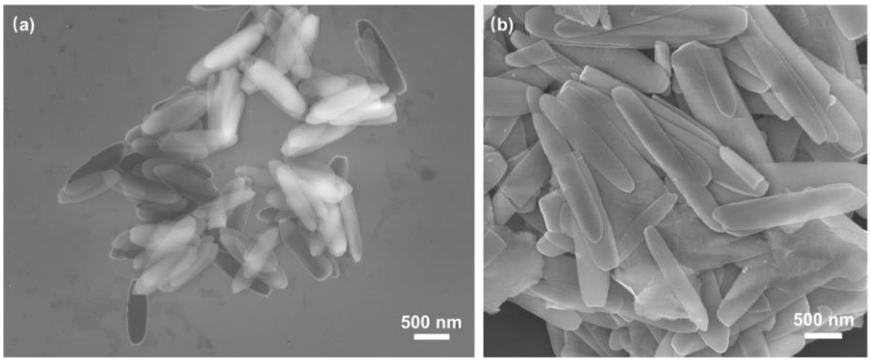

Supplementary Figure 17. SEM images of (a) $(CoMnNiCuZnBi)_2(OH)_2CO_3$ and (b) SC-MHEO-$(CoMnNiCuZnBi)_3O_4$.

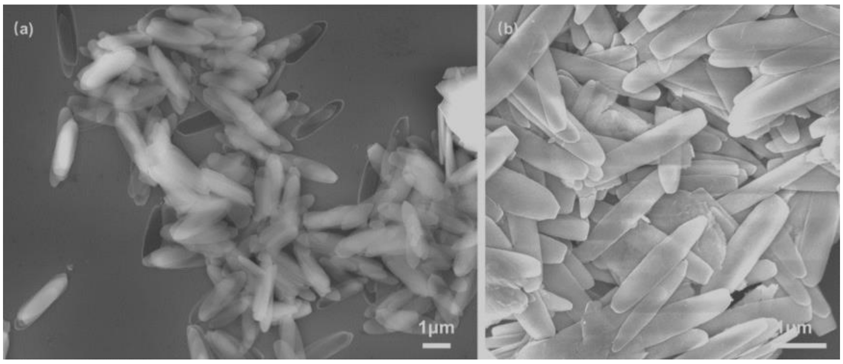

Supplementary Figure 18. SEM images of (a) $(CoMnNiCuZnFeBi)_2(OH)_2CO_3$ and (b) SC-MHEO-$(CoMnNiCuZnFeBi)_3O_4$.

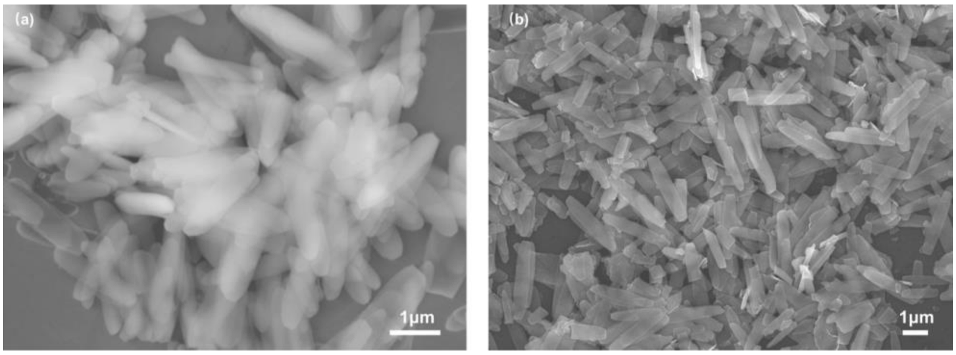

Supplementary Figure 19. SEM images of (a) $(CoMnNiCuZn)_2(OH)_2CO_3$ and (b) SC-MHEO-(CoMnNiCuZn)O.

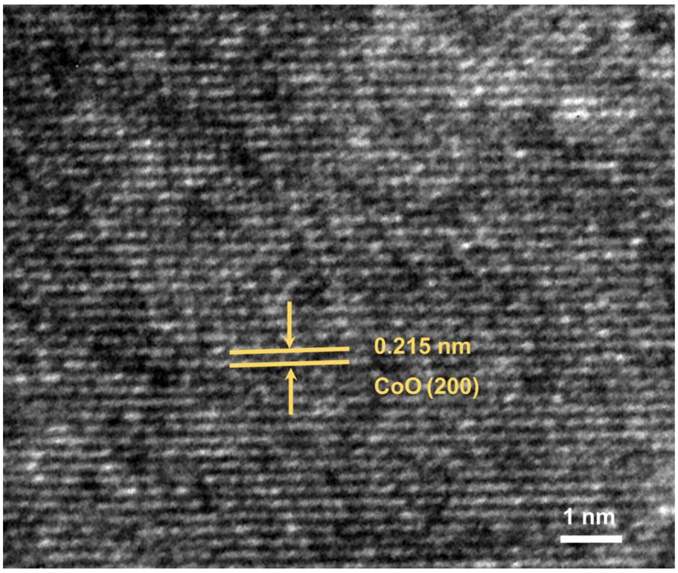

Supplementary Figure 20. High-resolution TEM image of SC-MHEO-(CoMnNiCuZn)O.

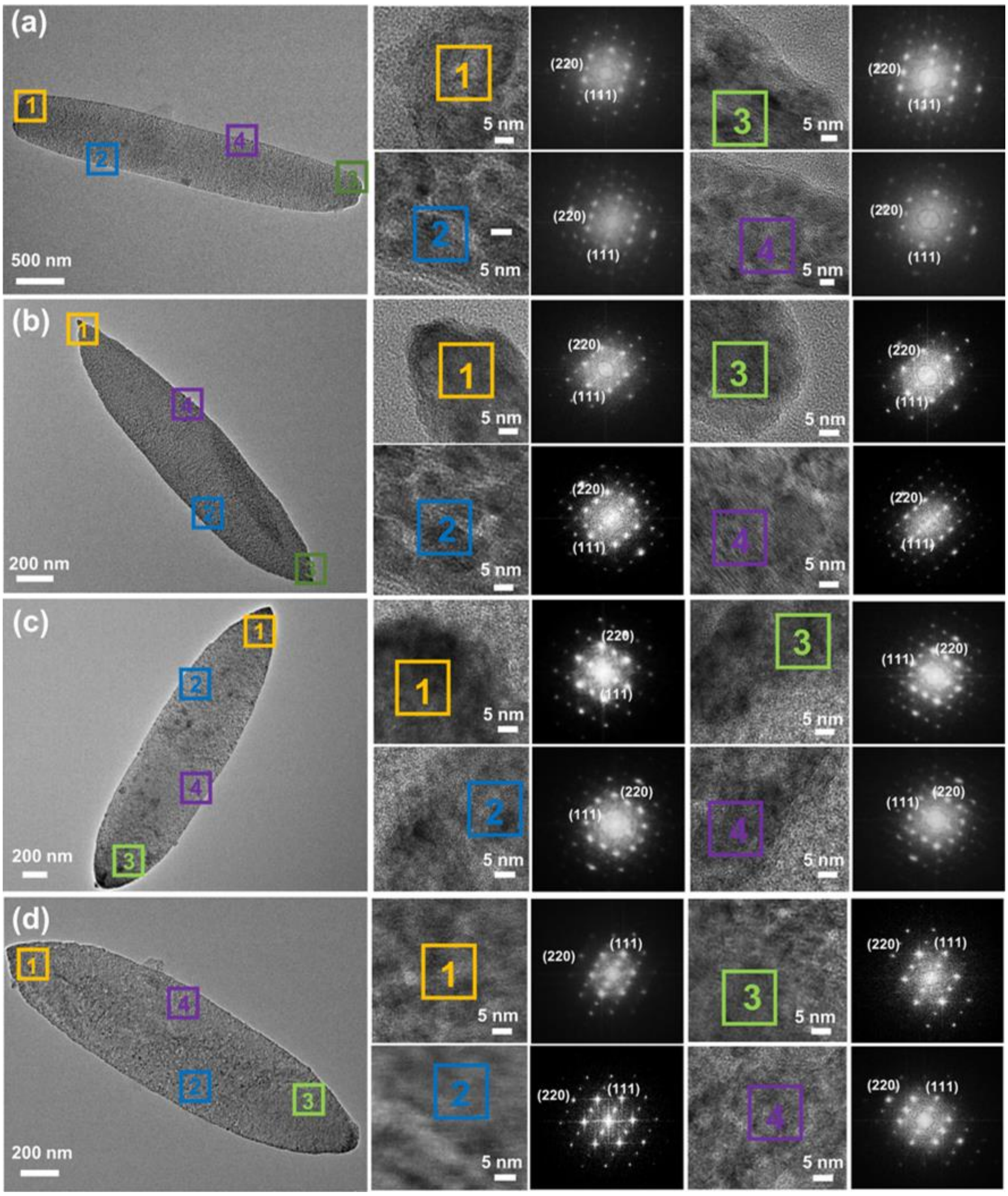

Supplementary Figure 21. TEM images and corresponding FT patterns of (a) SC-MHEO-$(CoNiMnCuFe)_3O_4$, (b) SC-MHEO-$(CoNiMnCuZnBi)_3O_4$, (c) SC-MHEO-$(CoNiMnCuZnBiFe)_3O_4$, and (d) SC-MHEO-(CoNiMnCuZn)O.

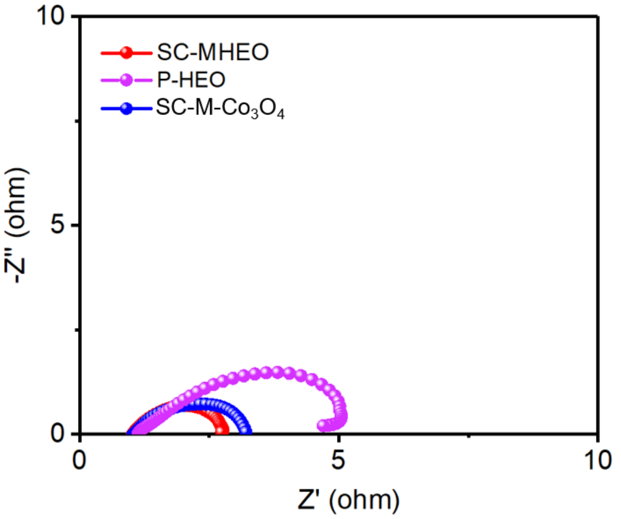

Supplementary Figure 22. EIS plots of SC-MHEO, P-HEO, and SC-M-Co_3O_4 collected at -1.485 V (vs RHE).

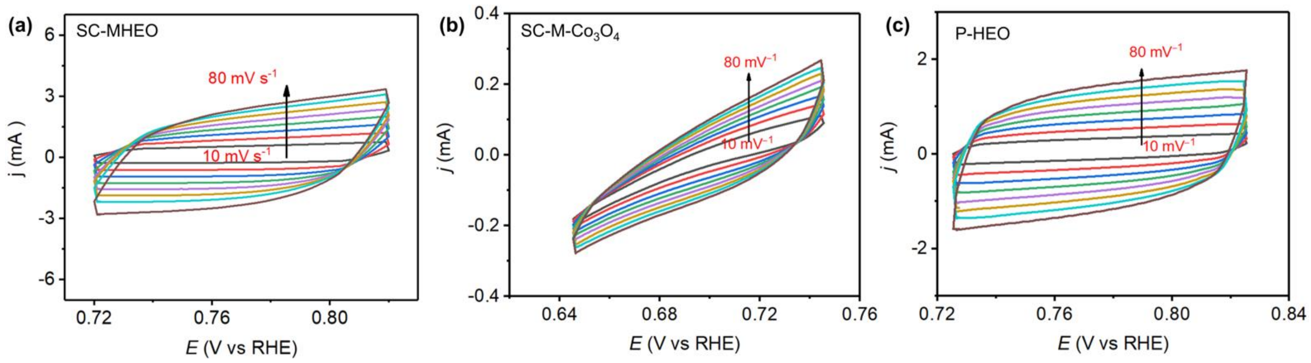

Supplementary Figure 23. CV curves of (a) SC-MHEO, (b) SC-M-Co_3O_4, and (c) P-HEO collected in different scan rates.

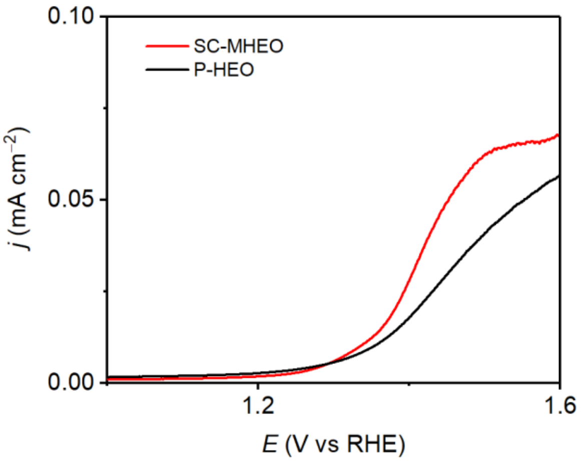

Supplementary Figure 24. ECSA-normalized polarization curves of SC-MHEO in 50 mM HMF.

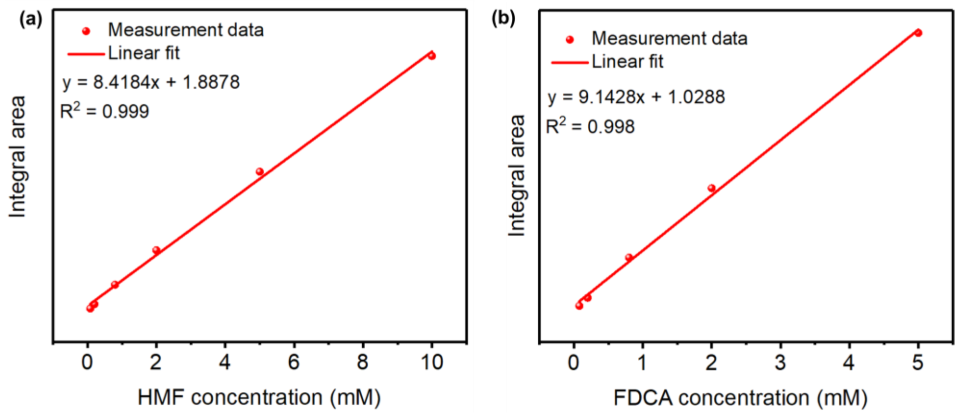

Supplementary Figure 25. The linear standard curves for the calculation of (a) HMF and (b) FDCA.

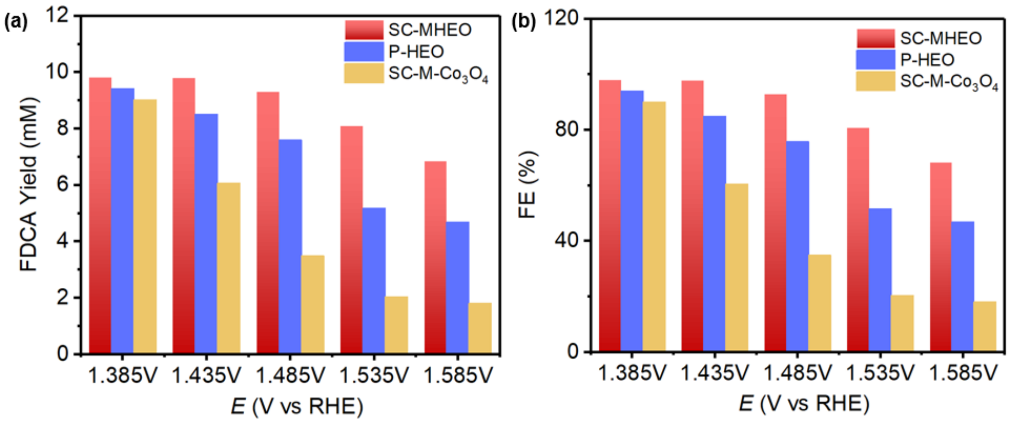

Supplementary Figure 26. (a) FDCA yields and (b) corresponding FE values of SC-MHEO, P-HEO, and SC-M-Co_3O_4 collected under different potentials.

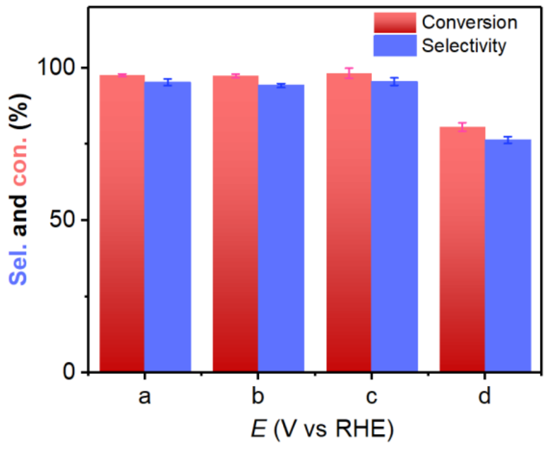

Supplementary Figure 27. Conversion of HMF and selectivity of FDCA for HMFOR electrocatalysized by SC-MHEO at 1.435 V (vs. RHE) (a: $(CoMnNiCuFe)_3O_4$; b: $(CoMnNiCuZnBi)_3O_4$; c: $(CoMnNiCuZnFeBi)_3O_4$; d: (CoNiMnCuZn)O (error bars are determined from five replicate trials at different potentials).

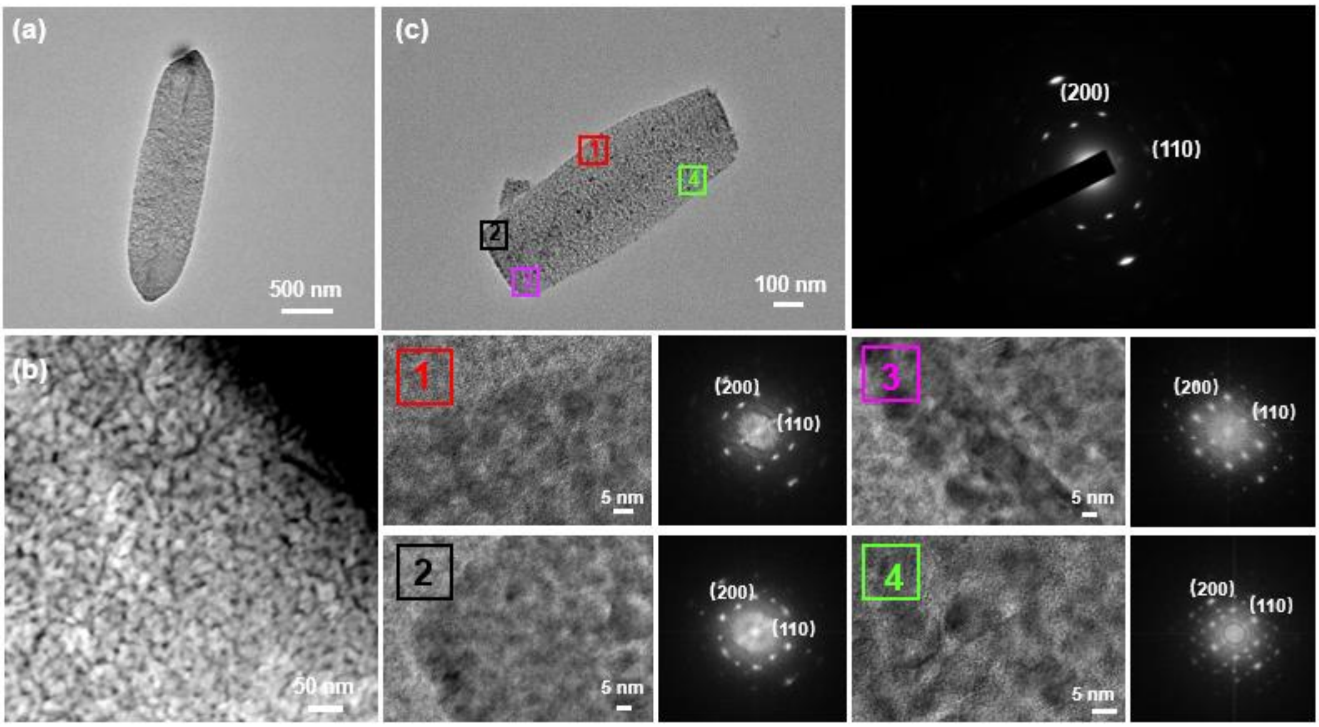

Supplementary Figure 28. (a) TEM and (b) high-magnification HAADF-STEM images, (c) TEM image and corresponding FT patterns of SC-MHEO after the stability test.

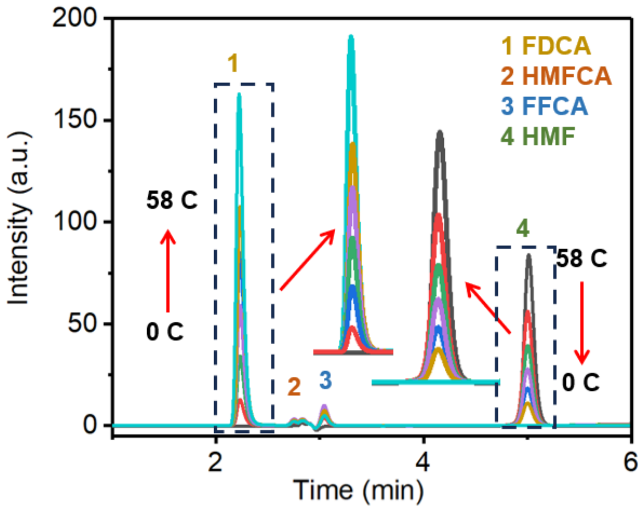

Supplementary Figure 29. HPLC chromatogram traces for various products at different C of SC-MHEO with 10 mM HMF at 1.435 V (vs. RHE).

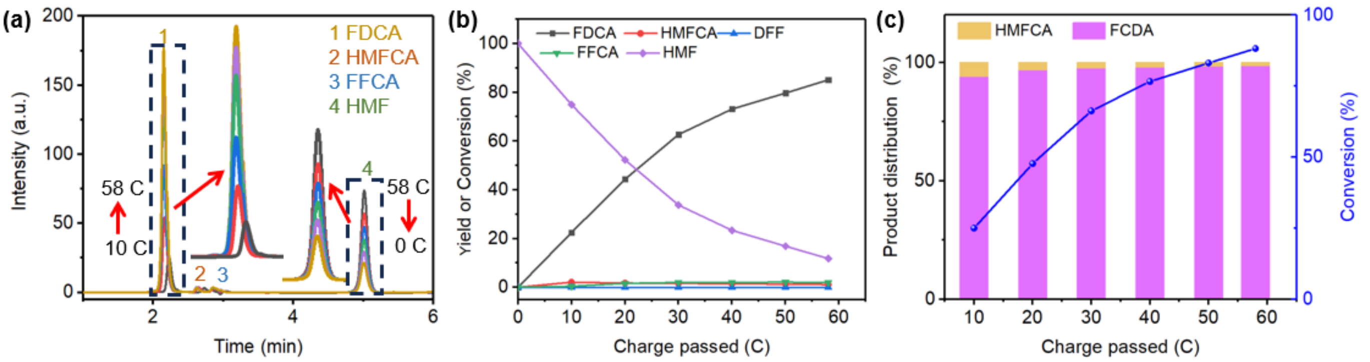

Supplementary Figure 30. (a) HPLC chromatogram traces for various products at different C of P-HEO with 10 mM HMF at 1.435 V (vs. RHE). (b) Concentration changes of HMF and its oxidation products, and (c) corresponding draw histograms summarized in (b).

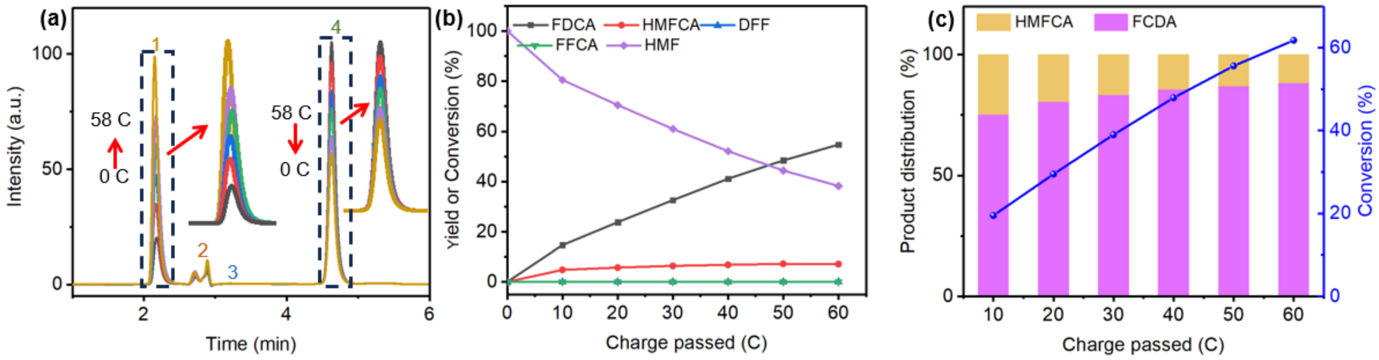

Supplementary Figure 31. (a) HPLC chromatogram traces for various products at different C of SC-M-Co_3O_4 with 10 mM HMF at 1.435 V (vs. RHE). Concentration changes of HMF and its oxidation products, and (c) corresponding draw histograms summarized in (b).

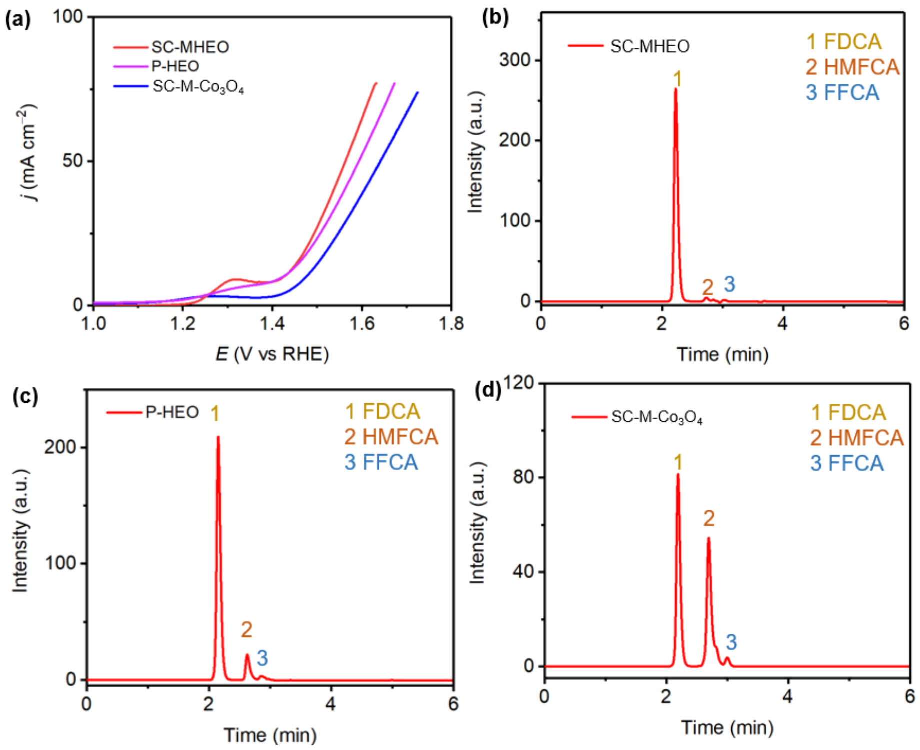

Supplementary Figure 32. (a) LSV curves of SC-MHEO, P-HEO, and SC-M-Co_3O_4 with 10 mM HMFCA. HPLC chromatogram traces for various products following an HMFCA electrochemical oxidation reaction of (b) SC-MHEO, (c) P-HEO and (d) SC-M-Co_3O_4.

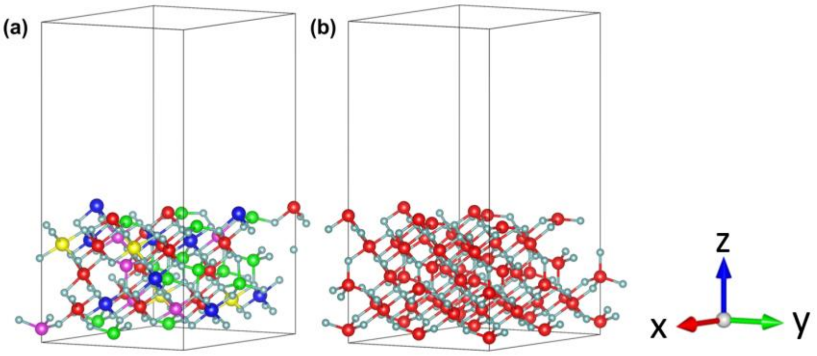

Supplementary Figure 33. Atomic structural models of (a) SC-MHEO and (b) SC-M-Co_3O_4.

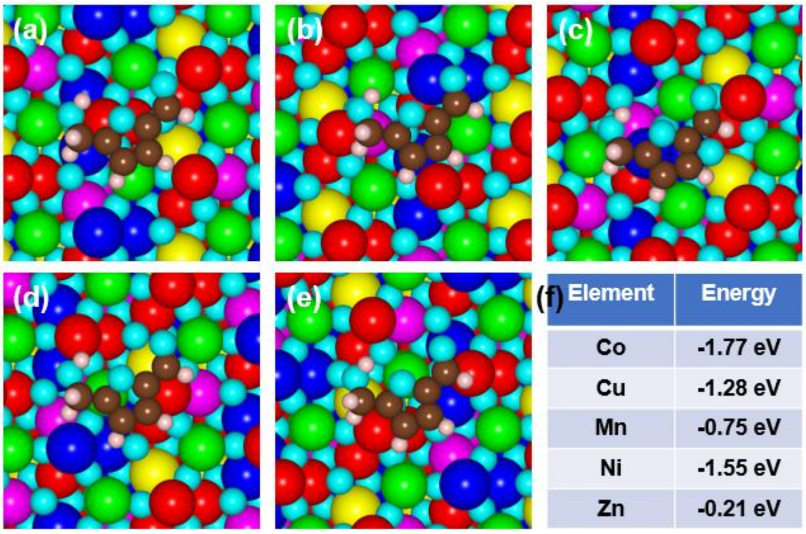

Supplementary Figure 34. Adsorption model structure of HMF at (a) Co, (b) Cu, (c) Ni, (d) Mn, and (e) Zn sites of SC-MHEO. (f) Calculated adsorption energies of Co, Cu, Ni, Mn, and Zn sites in the SC-MHEO structure.

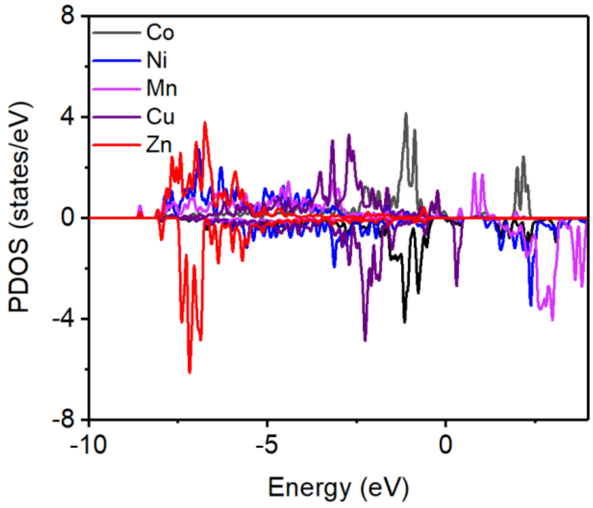

Supplementary Figure 35. PDOSs of SC-MHEO.

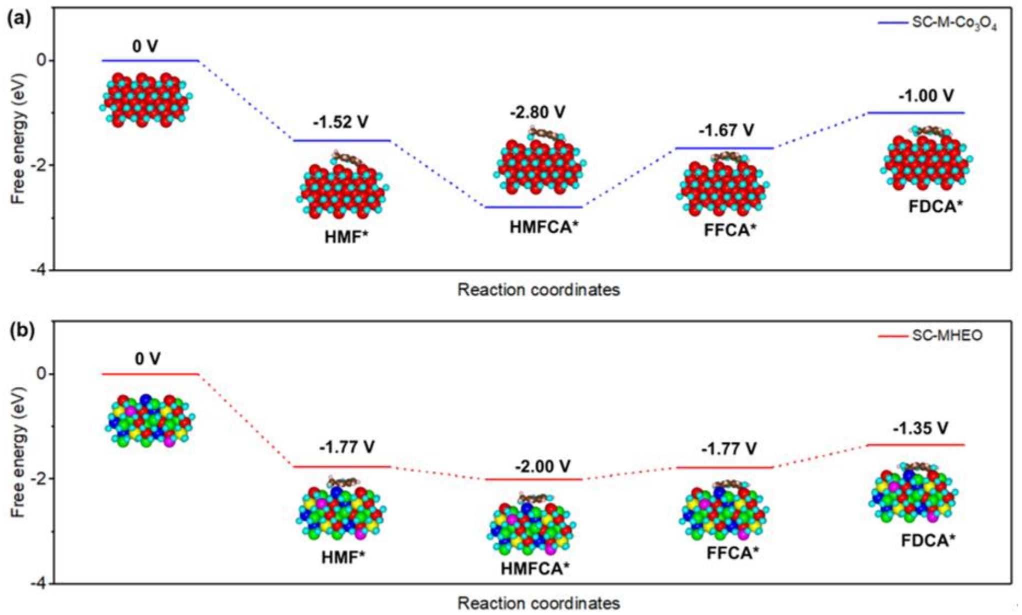

Supplementary Figure 36. Gibbs free energy diagrams and reaction paths for HMFOR electrocatalysis of (a) SC-MHEO and (b) SC-M-Co_3O_4.

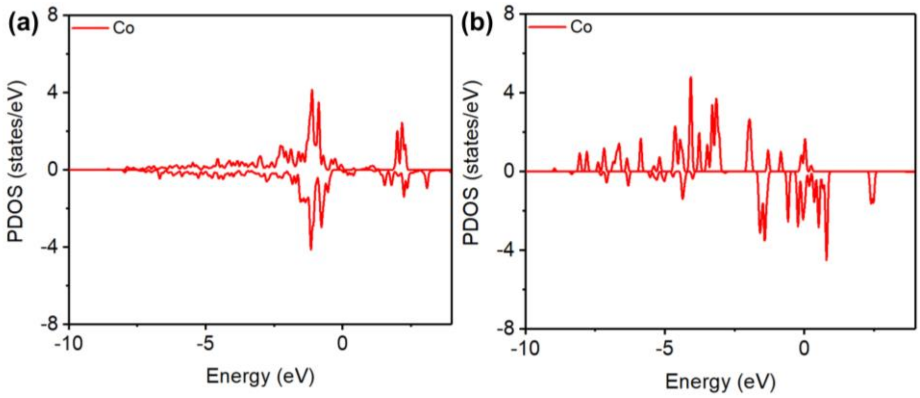

Supplementary Figure 37. Co PDOS of SC-MHEO and SC-M-Co_3O_4.

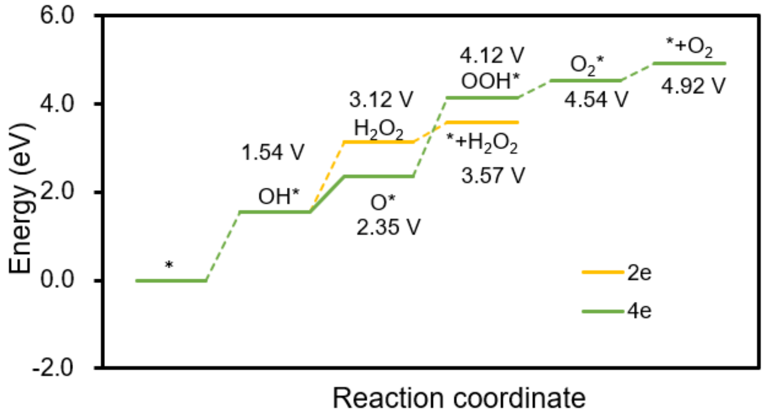

Supplementary Figure 38. Gibbs free energy diagrams and reaction paths for OER and WOR electrocatalysis of SC-MHEO.

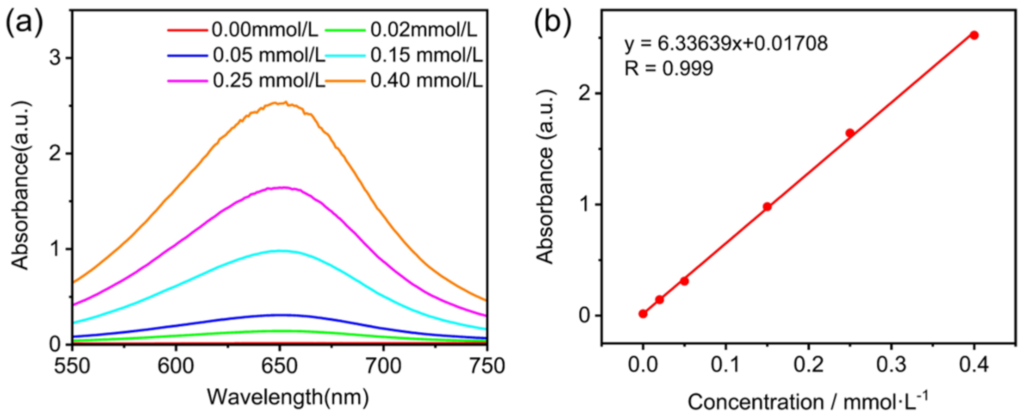

Supplementary Figure 39. Ammonia detection using an indophenol blue method (in 1.0 M KOH). (a) UV-Vis spectra of solutions with different ammonia concentrations. (b) The linear standard curve for the calculation of ammonia production.

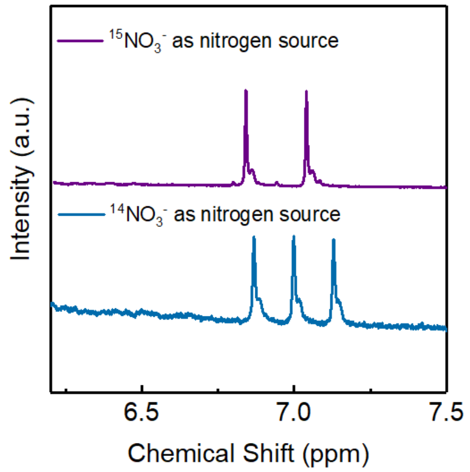

Supplementary Figure 40. ^{1}H NMR spectra of the electrolyte after electrocatalytic NO_3^-RR using $^{15}NO_3^-$ and $^{14}NO_3^-$ as the nitrogen source.

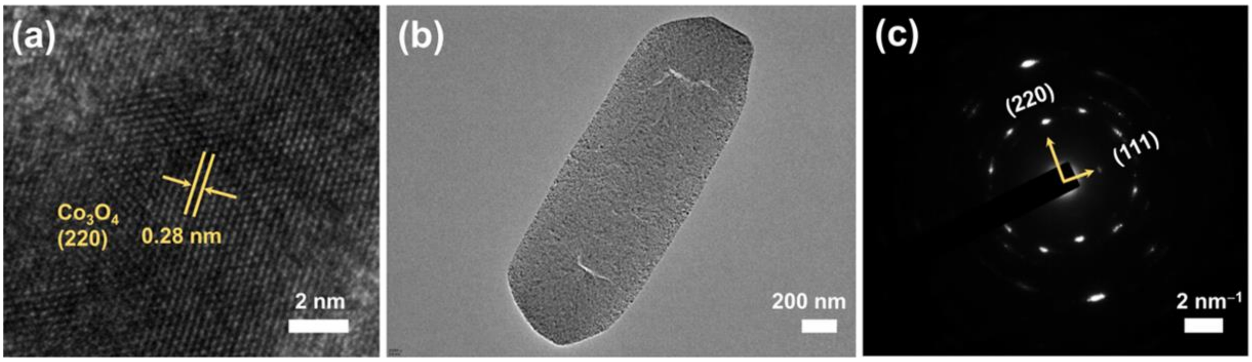

Supplementary Figure 41. (a) HR TEM image, (b) TEM image and (c) corresponding FT patterns of SC-MHEO after the stability test.

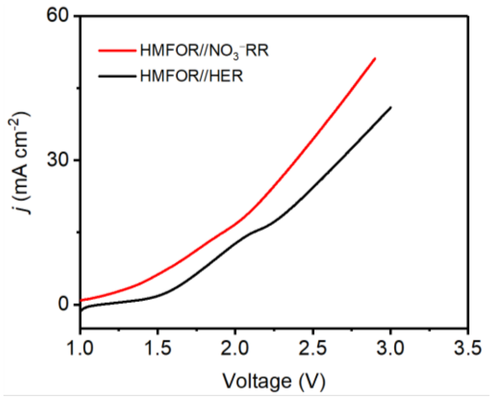

Supplementary Figure 42. LSV curves of SC-MHEO electrocatalyst in the two-electrode coupling system for (+) HMFOR|| NO_3^-RR (−) and (+) HMFOR|| HER (−).

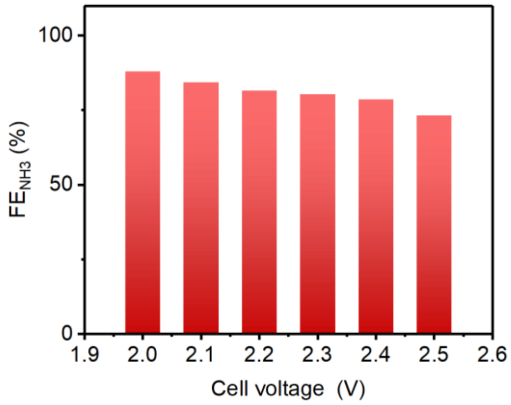

Supplementary Figure 43. FE_{NH3} of NO_3^--to-NH_3 electrocatalysis by SC-MHEO in two-electrode coupling system ((+) HMFOR|| NO_3^-RR (−)).

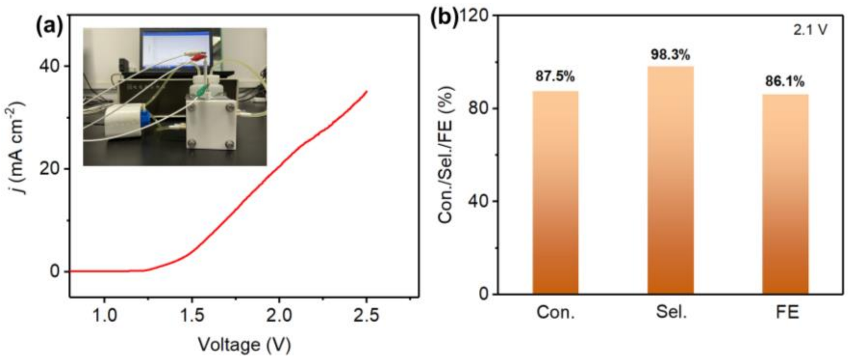

Supplementary Figure 44. (a) LSV curve of SC-MHEO electrocatalyst in the two-electrode coupling system continuous flow electrolyzer. Inset in (a) is the two-electrode coupling system continuous flow electrolyzer. (b) Conversion, selectivity and FE of SC-MHEO cathode in the two-electrode coupling system continuous flow electrolyzer at cell voltage of 2.1 V.

Supplementary Table 1. Element ratios of MHEO materials obtained from STEM EDS mappings and ICP-MS.

Catalyst	Element	Percentage (%)	
		from EDS mappings	from ICP-MS
SC-MHEO-$(CoMnNiCuZn)_3O_4$	Co	34.8	35.2
	Ni	28.1	28.3
	Mn	16.0	18.4
	Cu	13.2	11.3
	Zn	7.9	6.8
SC-MHEO-$(CoMnNiCuFe)_3O_4$	Co	41	38.2
	Cu	10.9	11.4
	Ni	25.5	26.8
	Mn	15	15.5
	Fe	7.6	8.1
SC-MHEO-$(CoMnNiCuZnBi)_3O_4$	Co	35.8	34.2
	Cu	8.3	10.2
	Ni	22.9	22.5
	Mn	16.5	18.3
	Zn	7.3	6.5
	Bi	9.2	8.3
SC-MHEO-$(CoMnNiCuZnFeBi)_3O_4$	Co	34.8	35.6
	Cu	8.7	9.1
	Ni	15.9	17.5
	Mn	14.5	15.9
	Zn	15	9.8
	Fe	5.8	6.2
	Bi	5.3	5.9
SC-MHEO-(CoMnCuNiZn)O	Co	40	39.1
	Cu	15.6	13.7
	Ni	24.4	25.8
	Mn	11.1	13.8
	Zn	8.9	7.6

Supplementary Table 2. Element ratios of mesoporous P-HEOs obtained from ICP-MS.

Element	Percentage (%)
**Co**	33.5
**Mn**	26.8
**Ni**	16.6
**Cu**	15.9
**Zn**	7.2

Supplementary Table 3. Calculated S_{config} values of SC-MHEO nanoplates.

Catalyst	S_{config}
**SC-MHEO-$(CoMnNiCuZn)_3O_4$**	**1.5R**
**SC-MHEO-$(CoMnNiCuFe)_3O_4$**	**1.6R**
**SC-MHEO-$(CoMnNiCuZnBi)_3O_4$**	**1.6R**
**SC-MHEO-$(CoMnNiCuZnFeBi)_3O_4$**	**1.8R**
**SC-MHEO-(CoMnCuNiZn)O**	**1.5R**

Supplementary Table 4. Summarized conversions of HMF and FE_{FDCA} values electrocatalyzed by SC-MHEO, P-HEO, and SC-M-Co_3O_4.

Catalyst	Potential (versus RHE)	Conversion (%)	FE_{FDCA} (%)
**SC-MHEO**	1.385	99.8	97.8
	1.435	99.3	97.7
	1.485	94.1	92.8
	1.535	90.6	80.7
	1.585	74.7	68.2
**P-HEO**	1.385	95.8	93.9
	1.435	87.2	84.9
	1.485	79.7	75.7
	1.535	54.1	51.5
	1.585	53.2	46.7
**SC-M-Co_3O_4**	1.385	98.5	89.9
	1.435	78.8	60.5
	1.485	41.2	34.9
	1.535	26.0	20.4
	1.585	24.1	18.0

Supplementary Table 5. Comparisons of performances on selective HMFOR to FDCA electrocatalyzed by SC-MHEO and other reported catalysts in alkaline condition.

Catalyst	Conversion (%)	FDCA yield (%)	FE_{FDCA} (%)	Reference
**SC-MHEO**	99.3	97.9	97.7	This work
**P-HEOs**	99	97.4	96.6	[10]
**Ni-Cu/NF**	100	>95	>95	[11]
**$NiOOH-Cu(OH)_2$**	100	98.3	98.3	[12]
**$Vo-Co_3O_4$**	-	91.9	95	[13]
**$Ni_{0.5}Co_{2.5}O_4$**	-	92.42	90.35	[14]
**MoO_2-FeP@C**	99.4	98	97.8	[15]
**$(FeCrCoNiCu)_3O_4$**	100	95	95	[10]
**$CuCo_2O_4$**	100	93.7	94	[16]
**VN**	90	94	90	[17]
**$Ni(OH)_2/NiOOH$**	99.8	96	96	[18]
**Ni_2P**	100	97	97	[19]
**$NiCo_2O_4$**	99.6	90.8	80	[20]
**CoNiFe LDH**	95.5	94.9	90	[21]
**Ni_3S_2**	100	98	98	[22]
**hp-Ni**	100	98	92	[23]
**CoP**	100	90	90	[24]
**Ni_3N@C**	-	98	99	[25]
**NiCoMn-LDH**	100	91.7	65	[26]
**$Mo-Ni_{0.85}Se$**	100	95	95	[27]
**Ni-CAT**	100	98.7	86.8	[28]

Supplementary References

[1] G. Kresse, J. Hafner, *Phys. Rev. B* 1993, **47**, 558.
[2] G. Kresse, J. Furthmüller, *Phys. Rev. B* 1996, **54**, 11169.
[3] G. Kresse, J. Furthmüller, *Comput. Mater. Sci.* 1996, **6**, 15.
[4] P. E. Blöchl, *Phys. Rev. B* 1994, **50**, 17953.
[5] G. Kresse, D. Joubert, *Phys. Rev. B* 1999, **59**, 1758.
[6] J. P. Perdew, Y. Wang, *Phys. Rev. B* 1992, **45**, 13244.
[7] J. P. Perdew, K. Burke, M. Ernzerhof, *Phys. Rev. Lett.* 1996, **77**, 3865.
[8] K. Momma, F. Izumi, *J. Appl. Crystallogr.* 2011, **44**, 1272.
[9] J. K. Nørskov, J. Rossmeisl, A. Logadottir, L. Lindqvist, J. R. Kitchin, T. Bligaard, H. Jónsson, *J. Phys. Chem. B.* 2004, **108**, 17886.
[10] K. Gu, D. Wang, C. Xie, T. Wang, G. Huang, Y. Liu, Y. Zou, L. Tao, S. Wang, *Angew. Chem. Int. Ed.* 2021, **60**, 20253.
[11] D. Chen, Y. Ding, X. Cao, L. Wang, H. Lee, G. Lin, W. Li, G. Ding, L. Sun, *Angew. Chem. Int. Ed.* 2023, **62**, e202309478.
[12] J. Woo, B. C. Moon, U. Lee, H.-S. Oh, K. H. Chae, Y. Jun, B. K. Min, D. K. Lee, *ACS Catal.* 2022, **12**, 4078.
[13] Y. Lu, T. Liu, C.-L. Dong, C. Yang, L. Zhou, Y.-C. Huang, Y. Li, B. Zhou, Y. Zou, S. Wang, *Adv. Mater.* 2022, **34**, 2107185.
[14] Y. Lu, T. Liu, Y.-C. Huang, L. Zhou, Y. Li, W. Chen, L. Yang, B. Zhou, Y. Wu, Z. Kong, Z. Huang, Y. Li, C.-L. Dong, S. Wang, Y. Zou, *ACS Catal.* 2022, **12**, 4242.
[15] G. Yang, Y. Jiao, H. Yan, Y. Xie, A. Wu, X. Dong, D. Guo, C. Tian, H. Fu, *Adv. Mater.* 2020, **32**, 2000455.
[16] Y. Lu, C.-L. Dong, Y.-C. Huang, Y. Zou, Z. Liu, Y. Liu, Y. Li, N. He, J. Shi, S. Wang, *Angew. Chem. Int. Ed.* 2020, **59**, 19215.
[17] S. Li, X. Sun, Z. Yao, X. Zhong, Y. Cao, Y. Liang, Z. Wei, S. Deng, G. Zhuang, X. Li, J. Wang, *Adv. Funct. Mater.* 2019, **29**, 1904780.
[18] B. J. Taitt, D.-H. Nam, K.-S. Choi, *ACS Catal.* 2019, **9**, 660.
[19] B. You, N. Jiang, X. Liu, Y. Sun, *Angew. Chem. Int. Ed.* 2016, **55**, 9913.
[20] M. J. Kang, H. Park, J. Jegal, S. Y. Hwang, Y. S. Kang, H. G. Cha, *Appl. Catal. B* 2019, **242**, 85.
[21] T. Arakawa, Y. Sato, M. Yamada, J. Takabe, Y. Moriwaki, N. Masamura, M. Kato, M. Aoyagi, T. Kamoi, T. Terada, K. Shimizu, N. Tsuge, S. Imai, S. Fushinobu, *ACS Catal.* 2020, **10**, 9.
[22] B. You, X. Liu, N. Jiang, Y. Sun, *J. Am. Chem. Soc.* 2016, **138**, 13639.
[23] B. You, X. Liu, X. Liu, Y. Sun, *ACS Catal.* 2017, **7**, 4564.
[24] N. Jiang, B. You, R. Boonstra, I. M. Terrero Rodriguez, Y. Sun, *ACS Energy Lett.* 2016, **1**, 386.
[25] N. Zhang, Y. Zou, L. Tao, W. Chen, L. Zhou, Z. Liu, B. Zhou, G. Huang, H. Lin, S. Wang, *Angew. Chem. Int. Ed.* 2019, **58**, 15895.
[26] B. Liu, S. Xu, M. Zhang, X. Li, D. Decarolis, Y. Liu, Y. Wang, E. K. Gibson, C. R. A. Catlow, K. Yan, *Green Chem.* 2021, **23**, 4034.
[27] C. Yang, C. Wang, L. Zhou, W. Duan, Y. Song, F. Zhang, Y. Zhen, J. Zhang, W. Bao, Y. Lu, D. Wang, F. Fu, *Chem. Eng. J.* 2021, **422**, 130125.
[28] Y. Zhang, N. Kornienko, *ChemSusChem* 2022, **15**, e202101587.
